# Immunohistochemical Expressions of Senescence-Associated Secretory Phenotype and Its Association With Immune Microenvironments and Clinicopathological Factors in Invasive Breast Cancer

**DOI:** 10.3389/pore.2021.1609795

**Published:** 2021-06-29

**Authors:** Min Hui Park, Jung Eun Choi, Jae-Ryong Kim, Young Kyung Bae

**Affiliations:** ^1^ Department of Pathology, Yeungnam University College of Medicine, Daegu, South Korea; ^2^ Department of Surgery, Division of Breast Surgery, Yeungnam University College of Medicine, Daegu, South Korea; ^3^ Department of Biochemistry and Molecular Biology, Yeungnam University College of Medicine, Daegu, South Korea

**Keywords:** immunohistochemistry, breast cancer, senescence, prognosis, markers

## Abstract

This study was undertaken to investigate immunohistochemical expression of the senescence-associated secretory phenotype (SASP) in invasive breast cancer (IBC) tissues and to determine relationships between SASP positivity and tumor microenvironments and the clinicopathological characteristics of IBC. Immunohistochemistry for senescence markers, that is, high mobility group box-1 (HMGB1), p16, p15, and decoy receptor 2 (DCR2), was performed in tissue microarrays of 1140 IBC samples. Cases positive for at least one of these four markers were considered SASP-positive. Relations between SASP and tumor characteristics, including immune microenvironments (stromal tumor-infiltrating lymphocytes [sTILs] density and numbers of intraepithelial CD103-positive [iCD103 + ] lymphocytes) and clinical outcomes were retrospectively evaluated. HMGB1, p16, p15, or DCR2 was positive in 6.7%, 26.6%, 21.1%, and 26.5%, respectively, of the 1,140 cases. Six hundred and five (53.1%) cases were SASP positive, and SASP positivity was significantly associated with histologic grade 3, high-sTIL and iCD103 + lymphocyte counts, absence of ER or PR, and a high Ki-67 index. Although SASP did not predict breast cancer-specific survival (BCSS) or disease-free survival (DFS) in the entire cohort, SASP positivity in luminal A IBC was associated with poor BCSS and DFS. However, patients with SASP-positive TNBC showed better survival than those with SASP-negative TNBC. In multivariate analysis, SASP positivity was an independent prognostic factor in both luminal A IBC and TNBC, although the effect on prognosis was the opposite. In conclusion, SASP would be involved in the modulation of immune microenvironments and tumor progression in IBC, and its prognostic significance depends on molecular subtype.

## Introduction

Internal and external stimuli can damage DNA, and when this damage cannot be repaired, cells undergo aging (senescence) or apoptosis. Senescent cells secrete inflammatory cytokines, chemokines, growth factors and matrix metalloproteinases (MMPs) and modulate their microenvironments, which is called senescence-associated secretory phenotype (SASP) and can take various cell-dependent forms [1–3].

Tumor cell senescence can be induced by oncogenes, radiotherapy, or chemotherapy, and senescent tumor cells that acquire SASP change tumor microenvironments. Furthermore, SASP factors are known to display anti-tumor effects by inducing the senescence of surrounding tumor cells through paracrine or autocrine mechanisms or by activating the host immune system to remove tumor cells [4–6]. However, SASP can also promote angiogenesis, proliferation, and epithelial-mesenchymal transition (EMT), which result in tumor progression and invasion [7]. Furthermore, cancer cells exhibiting SASP cause chronic inflammation, tissue fibrosis, and increase tumor cell drug resistance [4].

The senescence-associated markers that are usually used to identify SASP in tumor tissue samples include p16, p15, p53, ADP-ribosylation factor (ARF), p21, differentially expressed in chondrocytes protein 1 (DEC1), decoy receptor 2 (DCR2), and high mobility group box 1 (HMGB1**)** [8–12]. p16 inhibits cell division by regulating G1 to S transition, and loss of p16 function promotes cell cycle progression and is involved in the pathogenesis of several cancers [13,14]. In addition, p16 is upregulated during tissue aging [15] and has been considered a prognostic marker in cancer patients [16–18]. p15 regulates G1 progression and is upregulated by senescence-associated β-galactosidase (SA-β-Gal, a classic SASP marker) activation [9]. p15 also participates in growth arrest in Ras-transformed NIH 3T3 fibroblasts [19].

DCR2 is a TRAIL (tumor necrosis factor-related apoptosis-inducing ligand) receptor and inhibits TRAIL-induced apoptosis in response to chemotherapy [20]. HMGB1 is a member of the alarmin family and an important component of tissue damage signals and also inhibits apoptosis by reducing the functions of caspase-3 and -9 [21–23]. HMGB1 is translocated from nuclei to cytoplasm and extracellular space in senescent cells, and this promotes the release of SASP factors, such as interleukin-1β (IL-1β), IL-6, and MMP3 [8,10,11]. Therefore, loss of nuclear expression of HMGB1 has been used for identifying senescent cells [10,24,25].

Several studies have addressed SASP marker expression in Korea and overseas. p15, p16, and DCR2 were reported to be upregulated during prostate cancer progression [12], and DCR2 expression was increased in lung cancer cell lines treated with anticancer drugs [20]. In addition, HMGB1 upregulation has been observed in breast cancer [26], colon cancer [22] and gastrointestinal stromal tumor [27] tissues. However, few studies have shown combined expression of senescence markers and its association with clinicopathologic features in breast cancer [28].

In the present study, we used immunohistochemistry (IHC) to determine the expressions of senescence-associated markers in invasive breast cancer (IBC) tissues and then investigated the effects of SASP on tumor microenvironments, clinicopathological characteristics, and patient prognosis.

## Materials and Methods

### Collection of Breast Cancer Tissues and Clinicopathological Data

IBC tissue samples surgically resected at Yeungnam University Medical Center from 1995 to 2007 were included in this study. Patients that received neoadjuvant chemotherapy or with a microinvasive carcinoma were excluded. Clinicopathological information and follow-up data were collected from medical records including pathology reports. Breast cancer-specific survival (BCSS) was defined as time interval between surgical resection and death from -related cause or last follow-up. Disease-free survival (DFS) was defined as time interval between surgical resection and tumor relapse (locoregional recurrence or distant metastasis), death or last follow-up.

This study was approved by the Institutional Review Board (IRB) of Yeungnam University Medical Center (YUMC2019-10-002), which waived the requirement for informed consent.

### Tissue Microarrays and Immunohistochemical Staining for Senescence-Associated Markers

For this study, we used tissue microarray (TMA) blocks that have been used in our previous studies [29,30]. To briefly explain TMA construction, a pair of 1.5 mm-diameter tissue cores was retrieved from a representative tumor block using a manual tissue microarrayer (Quick-Ray^®^, Unitma, Seoul, Korea), and consecutively transferred to the recipient blocks (Unitma). A total of 38 TMA blocks representing 1518 IBC cases were created.

IHC staining for HMGB1, p16, p15, and DCR2 was performed on TMA sections using a Benchmark XT immunostainer (Ventana Medical Systems) as described in Table 1. All IHC slides were interpreted by two observers (YKB and MHP) under a multi-headed microscope. HMGB1 was ubiquitously expressed in nuclei of most cells including non-neoplastic and cancer cells. Therefore, loss of nuclear staining or faint nuclear staining compared with internal control (non-neoplastic epithelial cells, fibroblasts, endothelial cells, or inflammatory cells) in >50% of tumor cells was considered positive for HMGB1. Immunoreactivity for p16, p15, or DCR2 was not observed in both nuclei and cytoplasm of normal breast epithelial cells. For these markers, intensities of cytoplasmic staining and proportions of immunoreactive tumor cells were evaluated. Staining intensities were scored as follows: 0 (no staining), 1 (weak), 2 (moderate), and 3 (strong). Proportions of immunoreactive tumor cells were expressed as percentages. IHC scores were generated by multiplying intensity scores by percentages of immunoreactive tumor cells, which resulted in a range of 0–300. We used 75th percentile IHC scores as cut-off values to define positivity for p16, p15, and DCR2. Cases positive for at least one of the four markers were considered SASP-positive tumors.

**TABLE 1:** Antibodies and staining conditions used in the study.

Antibody	Source	Clone	Dilution	Antigen retrieval	Incubation time
HMGB1	Abcam	EPR3507	1:400	Mild^a^, CC1	40 min, RT
p15	Abcam	Polyclonal	1:200	Autoclave, 10min	10 h, 4°C
p16	Ventana	E6H4	Prediluted	Standard^b^, CC1	16 min, 37°C
DCR2	Abcam	EPR3588(2)	1:250	Standard^b^, CC1	40 min, RT
CD103	Abcam	EPR4166(2)	1:500	Mild^a^, CC1	40 min, RT

CC1, cell conditioning 1 solution; DCR2, decoy receptor 2; HMGB1, high mobility group box-1; RT, room temperature.

a Mild antigen retrieval condition was performed for 30 min at 100°C.

b The standard condition was 60 min at 100°C.

These procedures were performed using a Benchmark^®^ XT autoimmunostainer.

### Molecular Classification of Invasive Breast Cancer

In order to apply consistent criteria on determining estrogen receptor (ER), progesterone receptor (PR) and human epidermal growth factor receptor 2 (HER2) statuses, we repeated ER, PR, and HER2 studies on TMA sections, as described in our previous study [31] and their results were interpreted according to the latest guidelines [32,33].

Ki-67 indices were reported at diagnosis and expressed as percentages of positive cells per 500–1,000 tumor cells.

Definition used for surrogate molecular subtypes of IBC was as follows [34]; luminal A (ER-positive/PR-positive/HER2-negative/Ki-67 ≤ 20%), HER2-negative luminal B1 (ER-positive/HER2-negative/Ki-67 > 20% or ER-positive/HER2-negative/PR-negative or low), HER2-positive luminal B2 (ER-positive/HER2-positive/any Ki-67/any PR), HER2-positive (ER-negative/PR-negative/HER2-positive), or triple-negative (TNBC) (ER-negative/PR-negative/HER2-negative). A low PR status was defined as an Allred score of <5.

### Tumor Microenvironment: Stromal Tumor-Infiltrating Lymphocyte and Intraepithelial CD103-Positive (iCD103+) Lymphocyte Measurements

The stromal tumor-infiltrating lymphocyte (sTIL) density and intraepithelial CD103 − positive (iCD103 + ) lymphocyte count for each case were obtained from our previous studies conducted in the same IBC cohort [35,30]. Brief descriptions of the research methods are as follows. sTIL densities were defined as percentages of total intratumoral stromal areas infiltrated with lymphocytes and plasma cells and measured on a whole section HandE slide for each case [36]. Under low magnification observations (X100), average sTIL densities were presented as; 0–1%, 2–5%, 6–10%, 11%–20%, 21–30%, or further 10% increments [35]. iCD103 + lymphocytes were defined as CD103 + lymphocytes in tumor cell nests or CD103 + lymphocytes adhering to tumor cells when tumors exhibited highly infiltrative growth [37,38]. IHC for CD103 [EPR4166(2), 1:500, Cambridge, United Kingdom] was performed using TMA sections [30], and stained slides were scanned using an Aperio CS2 slide scanner (Leica Biosystems, Nussloch, Germany). In an area captured at × 200 magnification (0.45 mm^2^) with the highest CD103 + lymphocyte density, total numbers of iCD103 + lymphocytes were manually counted and converted into numbers per 1 mm^2^.

### Statistical Analysis

Statistical analysis was conducted using SPSS (Version 23.0 for Windows, IBM, Armonk, NY, United States). The significance of correlations between SASP and patient characteristics was determined using chi-square test. Survival curves were plotted using the Kaplan-Meier method and the significance of survival differences between groups was determined using the log-rank test. Variables found to be significant by univariate analyses were subjected to Cox regression proportional hazard analysis. Adjusted hazard ratios and associated 95% confidence intervals were calculated for each variable. A *p* value of <0.05 was considered statistically significant.

## Results

### Clinicopathological Characteristics of Cases

Of the 1518 IBC samples included in the TMAs, only 1,140 yielded informative IHC results for the four SASP markers (HMGB1, p16, p15, and DCR2). The other 378 samples were exhausted by prior use, lost while performing immunosistochemical staining, or included ductal carcinoma *in situ*, extensive necrosis, or non-neoplastic tissue rather than viable invasive tumor.

Patients ranged in age from 20 to 86 years (mean, 48 years), and tumor sizes were 0.5–11.0 cm (mean, 2.4 cm). Axillary lymph node metastasis was present in 544 (47.8%), and lymphovascular invasion (LVI) in 600 (52.6%). Histological grades were 1 in 178 (15.6%), 2 in 326 (28.6%), and 3 in 636 (55.8%) patients **(** Table 2 **)**.

**TABLE 2:** Relationships between the senescence-associated secretory phenotype (SASP) and clinicopathological characteristics in patients with invasive breast carcinoma.

Characteristics	All patients, N (%)			*p*-value	Luminal A, N (%)			*p*-value	Triple-negative, N (%)			*p*-value
	No	SASP (−)	SASP (+)		No	SASP (−)	SASP (+)		No	SASP (−)	SASP (+)		
Age				0.239				0.245				0.01*
< 50	713	325 (45.6)	388 (54.4)		207	133 (64.3)	74 (35.7)		153	22 (14.4)	131 (85.6)	
≥ 50	427	210 (49.2)	217 (50.8)		96	55 (57.3)	41 (42.7)		94	26 (27.7)	68 (72.3)	
Tumor size				0.1				0.951				**0.03**
≤ 2 cm	565	279 (49.4)	286 (50.6)		193	120 (62.2)	73 (37.8)		101	13 (12.9)	88 (87.1)	
> 2 cm	575	256 (44.5)	319 (55.5)		110	68 (61.8)	42 (38.2)		146	35 (24)	111 (76)	
LN metastasis^a^				0.163				0.31				0.088
Absent	594	267 (44.9)	327 (55.1)		167	108 (64.7)	59 (35.3)		155	25 (16.1)	130 (83.9)	
Present	544	267 (49.1)	277 (50.9)		134	79 (59)	55 (41)		92	23 (25)	69 (75)	
LVI				0.445				**0.008**				**0.019**
Absent	540	247 (45.7)	293 (54.3)		148	103 (69.6)	45 (30.4)		150	22 (14.7)	128 (85.3)	
Present	600	288 (48)	312 (52)		155	85 (54.8)	70 (45.2)		97	26 (26.8)	71 (73.2)	
Histologic grade				**<0.001**				0.113				**0.001**
1 and 2	504	301 (59.7)	203 (40.3)		267	170 (63.7)	97 (36.3)		22	10 (45.5)	12 (54.5)	
3	636	234 (36.8)	402 (63.2)		36	18 (50)	18 (50)		225	38 (16.9)	187 (83.1)	
Stromal TILs^b^				**<0.001**				0.117				0.981
Low (≤ 5%)	798	423 (53)	375 (47)		274	174 (63.5)	100 (36.5)		108	21 (19.4)	87 (80.6)	
High (> 5%)	337	110 (32.6)	227 (67.4)		27	13 (48.1)	14 (51.9)		138	27 (19.6)	111 (80.4)	
iCD103 + lymphocyte^c^				**<0.001**				0.869				**0.042**
Low (< 38/ mm^2^)	820	417 (50.9)	403 (49.1)		252	153 (60.7)	99 (39.3)		122	29 (23.8)	93 (76.2)	
High (≥38/mm^2^)	212	58 (27.4)	154 (72.6)		12	7 (58.3)	5 (41.7)		106	14 (13.2)	92 (86.8)	
ER				**<0.001**								
Positive	764	434 (56.8)	330 (43.2)									
Negative	376	101 (26.9)	275 (73.1)									
PR				**<0.001**								
Positive	645	368 (57.1)	277 (42.9)									
Negative	495	167 (33.7)	328 (66.3)									
HER2				0.882								
Negative	912	427 (46.8)	485 (53.2)									
Positive	228	108 (47.4)	120 (52.6)									
Ki-67 index^d^				**<0.001**								
≤ 20%	444	258 (58.1)	186 (41.9)									
> 20%	687	275 (40)	412 (60)									
Molecular subtype				**<0.001**								
Luminal A	303	188 (62)	115 (38)									
Luminal B1	362	191 (52.8)	171 (47.2)									
Luminal B2	100	55 (55)	45 (45)									
HER2-positive	128	53 (41.4)	75 (58.6)									
TNBC	247	48 (19.4)	199 (80.6)									

ER, estrogen receptor; HER2, human epidermal growth factor receptor 2; iCD103 + lymphocyte, intraepithelial CD103-positive lymphocyte; LN, lymph node; LVI, lymphovascular invasion; TILs, tumor-infiltrating lymphocytes; TNBC, triple-negative breast cancer; PR, progesterone receptor.

a Two patients did not undergo sentinel lymph node biopsy or axillary lymph node dissection.

b Stromal TILs densities were not available in five patients.

c iCD103 + lymphocyte counts were missing in 108 patients.

d Nine patients did not have a Ki-67 labeling index in their pathology reports.

*p-values in bold, statistically significant.

**FIGURE 1:**
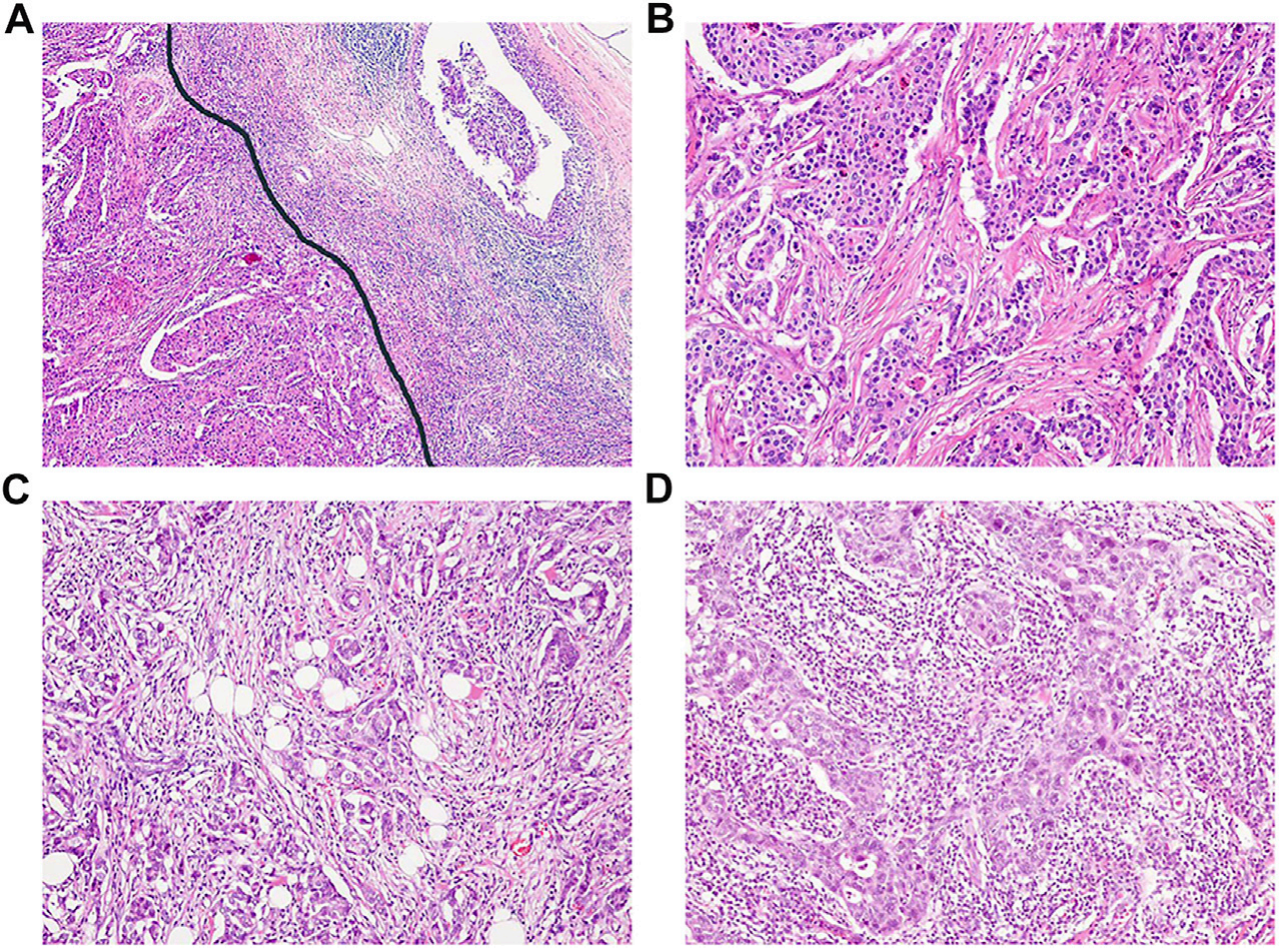
Representative cases with different stromal tumor-infiltrating lymphocyte (sTIL) densities. **(A)** sTIL densities were measured inside invasive tumor borders (left of the black line) ( × 40). Microscopic findings showing sTIL densities of **(B)** 1%, **(C)** 30%, and **(D)** 80% (b-d, × 100).

sTIL density could be obtained in 1,135 of 1,140 cases and ≤1% in 554 (48.8%) cases, 2–5% in 244 (21.5%), 6–10% in 125 (11%), 11–20% in 76 (6.7%), 21–30% in 40 (3.5%), 31–40% in 26 (2.3%), 41–50% in 27 (2.4%), 51–60% in 20 (1.8%), 61–70% in 13 (1.1%), 71–80% in 8 (0.7%), and 81–90% in 2 (0.2%) **(** Figure 1 **)**. For statistical analysis, cases were divided into low- and high-sTIL groups, based on our previous study [35]. As a result, 798 (70.3%) cases were allocated to the low-sTIL (≤5%) group and 337 (29.7%) to the high-sTIL (>5%) group.

**FIGURE 2:**
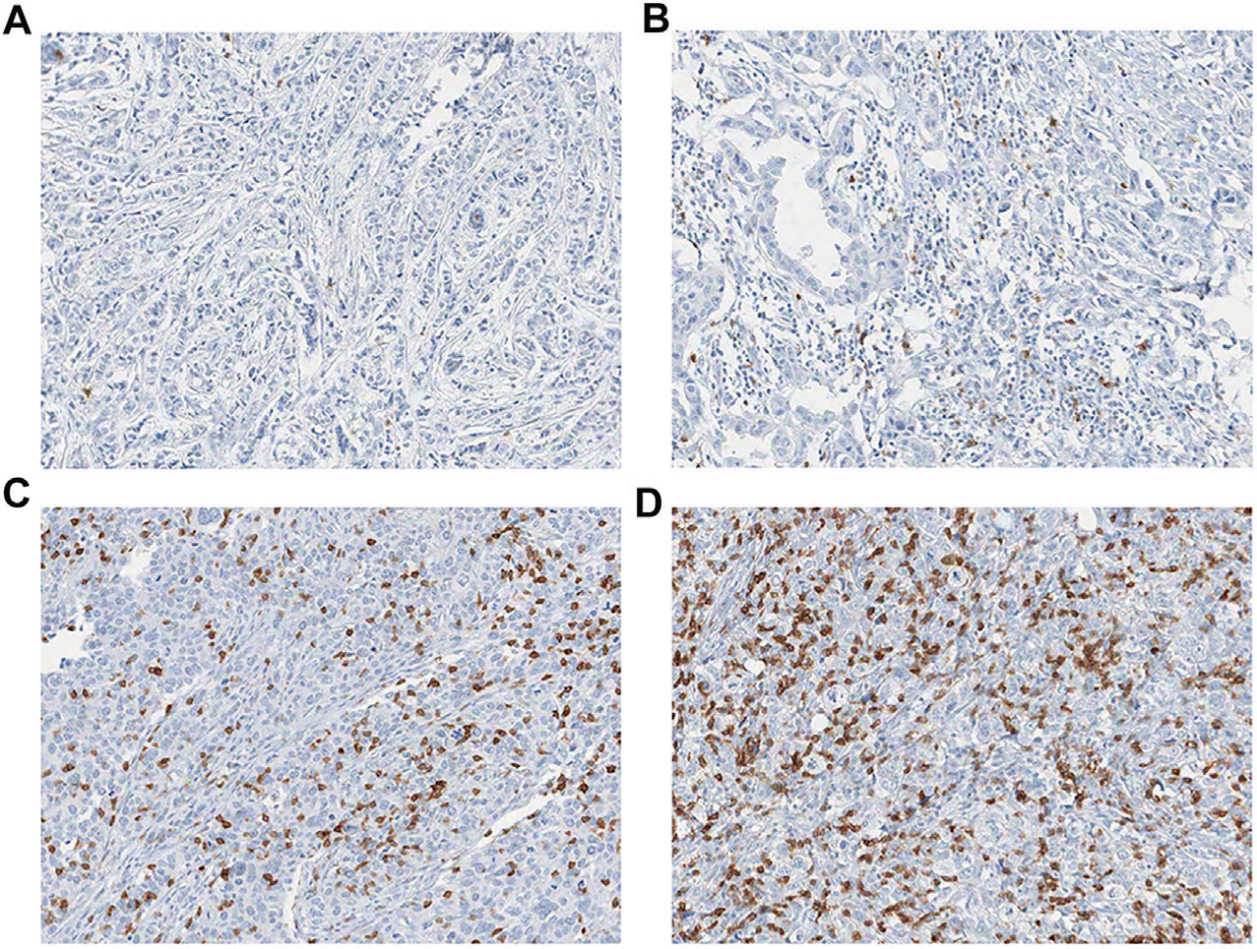
Representative images of **(A–B)** low (<38/ mm2) and **(C–D)** high (≥ 38/ mm2) densities of intraepithelial CD103 + (iCD103 + ) lymphocytes (all, × 200).

iCD103 + lymphocyte counts were available in 1,032 of 1,140 cases and ranged from 0 to 1,111 per mm^2^ of tumor area **(** Figure 2 **)**. Cases were dichotomized into high (≥38/ mm^2^) and low (<38/ mm^2^) iCD103 + groups, as we previously described [30]. As a result, 820 (79.5%) belonged to the low-iCD103 + group and 212 (20.5%) to the high-iCD103 + group.

Regarding biologic markers of IBC, ER was positive in 764 (67%), PR was positive in 645 (56.6%) and HER2 positivity (protein overexpression or gene amplification) was observed in 228 (20%). Ki-67 index was available in 1,131 of 1,140 cases and >20% in 687 (60.7%). As regards molecular subtypes, 303 (26.6%) cases were classified as luminal A, 362 (31.8%) as luminal B1, 100 (8.8%) as luminal B2, 128 (11.2%) as HER2-positive, and 247 (21.7%) as triple-negative (TNBC) **(** Table 2 **)**.

Of the 1,140 patients, 444 (38.9%) received breast-conserving surgery and 696 (61.1%) mastectomy. For adjuvant therapy, 1,003 (88%) received chemotherapy, 545 (47.8%) received radiation, and 772 (67.7%) received hormone therapy. None of the HER2-positive IBC patients received trastuzumab because trastuzumab as an adjuvant regimen was approved in Korea in 2010. The median follow-up period was 133 months (range, 1–277 months), and 212 (18.6%) recurrences and 223 (19.6%) deaths had occurred including 144 breast cancer-related deaths and 79 deaths from other causes.

### Expressions of Senescence-Associated Markers and Relationships Between SASP and Clinicopathological Characteristics

**FIGURE 3:**
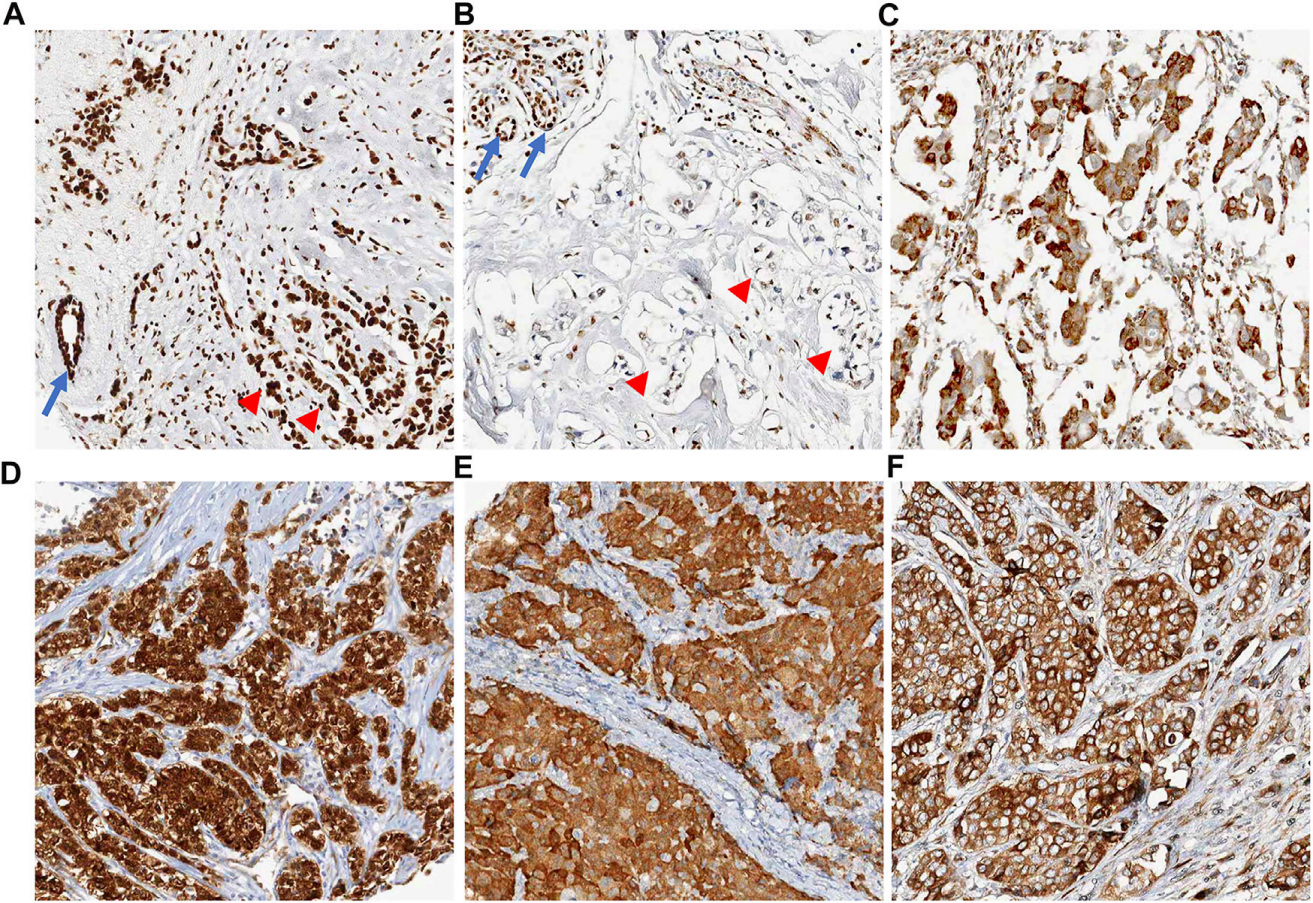
Representative images of positive cases for senescence-associated markers. **(A)** HMGB1 is ubiquitously expressed in all nuclei of non-neoplastic cells (arrow, normal duct) and cancer cells (arrow heads). There is absence of cytoplasmic staining. **(B)** Positive case for HMGB1. Tumor cells show faint nuclear staining or loss of nuclear staining without cytoplasmic staining (arrow heads). In left upper corner of the image, normal mammary glandular cells show strong nuclear staining for HMGB1 (arrows, internal control). **(C)** Positive case for HMGB1. This case shows faint nuclear staining with perinuclear cytoplasmic staining for HMGB1. **(D)** Positive case for p16. Tumor cells show diffuse immunoreactivity for p16 in both cytoplasm and nuclei. **(E)** Positive case for p15. Most of the nuclei of tumor cells was not stained, only cytoplasm was stained. **(F)** Positive case for DCR2. Tumor cells show diffuse cytoplasmic staining for DCR2.

Seventy-six (6.7%) cases showed loss of nuclear staining or faint nuclear staining for HMGB1 with or without cytoplasmic staining, therefore they were considered positive for HMGB1. IHC scores corresponding to 75th percentiles were 30, 10, and 10 for p16, p15, and DCR2, respectively. Of the 1,140 cases, 303 (26.6%), 240 (21.1%), and 302 (26.5%) were positive for p16, p15, and DCR2, respectively **(** Figure 3 **)**. As a total, 605 (53.1%) were positive for at least one senescence-associated marker (362 for one marker, 176 for two markers, 64 for three markers, and three for all four markers) and were regarded as SASP-positive cases. Among the five molecular subtypes, SASP positivity was found in 38% of luminal A cases, 47.2% of luminal B1 cases, 45% of luminal B2 cases, 58.6% of HER2-positive cases, and 80.6% of TNBC cases.

Among the 1,140 cases, SASP positivity was significantly associated with histologic grade (*p* < 0.001), sTIL density (*p* < 0.001), iCD103 + lymphocyte count (*p* < 0.001), ER negativity (*p* < 0.001), PR negativity (*p* < 0.001), and a high Ki-67 index (*p* < 0.001) but not with clinicopathological variables, such as age, tumor size, lymph node metastasis, LVI, or HER2 status **(** Table 2 **)**. Furthermore, SASP positivity was significantly greater in HER2-positive IBC and TNBC **(** Table 2 **)**.

When correlations between SASP and clinicopathological features were analyzed for the surrogate molecular subtypes: in the luminal A subtype, SASP was found to be significantly associated with LVI (*p* = 0.008); in the luminal B1 subtype, SASP was significantly associated with an age of < 50 (*p* = 0.023), a tumor size of >2 cm (*p* = 0.05), and high sTILs density (*p* = 0.024); in the luminal B2 subtype, SASP was not associated with any clinicopathological variable; in the HER2-positive subtype, SASP was significantly associated with high iCD103 + lymphocyte count (*p* = 0.045); and in the TNBC subtype, SASP was significantly associated with an age of <50 (*p* = 0.01), a tumor size of ≤2 cm (*p* = 0.03), absence of LVI (*p* = 0.019), high histologic grade (*p* = 0.001), and a high-iCD103 + lymphocyte count (*p* = 0.042) **(** Table 2 **)**.

### Prognostic Significance of SASP

**FIGURE 4:**
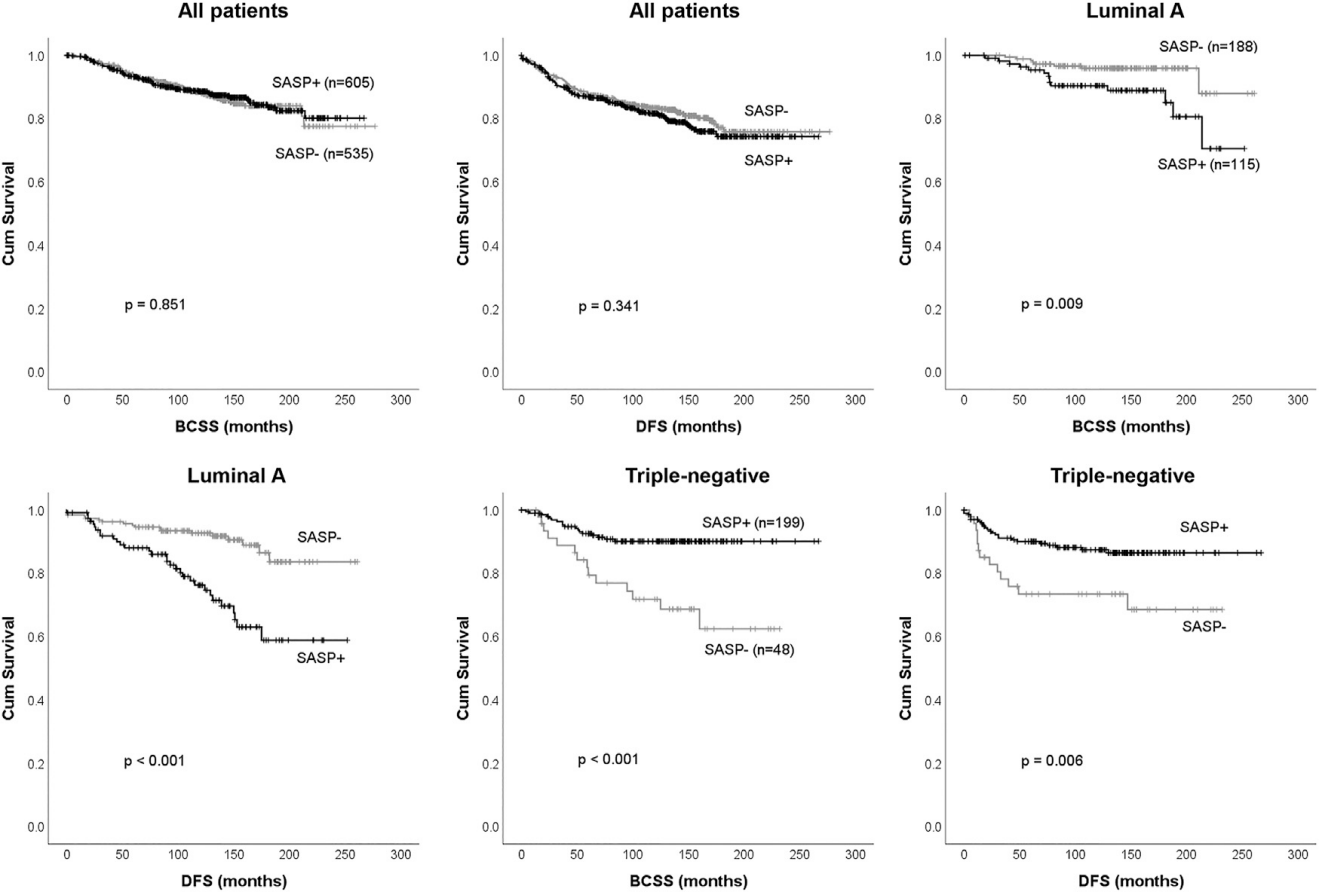
Survival analysis according to presence or absence of SASP. Breast cancer-specific survival (BCSS), and disease-free survival (DFS) for all 1,140 breast cancer patients **(A-B)** and patients with the luminal A subtype **(C-D)** or triple-negative breast cancer **(E-F)**.

Among the 1,140 cases, BCSS and DFS according to presence or absence of SASP were similar (*p* = 0.851 and *p* = 0.341, respectively) **(** Figures 4A,B **)**. We performed subgroup analyses to explore the association between SASP and prognosis in different surrogate molecular subtypes. Of the 303 patients with luminal A subtype, those with a SASP-positive tumor had significantly shorter BCSS (*p* = 0.009) and DFS (*p* < 0.001) **(** Figures 4C,D **)**, whereas in TNBC patients, SASP was significantly associated with better BCSS (*p* < 0.001) and DFS (*p =* 0.006) **(** Figures 4E,F **)**. However, no such relations were observed for luminal B1, B2 or HER2-positive subtypes.

Multivariate analyses showed SASP independently predicted poor BCSS (HR, 2.884; CI, 1.204–6.909; *p =* 0.017) and DFS (HR, 3.119; CI, 1.731–5.618; *p <* 0.001) for the luminal A subtype, but on the contrary it was an independent predictor of better BCSS (HR, 0.355; CI, 0.167–0.757; *p* = 0.007) and DFS (HR, 0.437; CI, 0.215–0.889; *p* = 0.022) in TNBC **(** Table 3 **)**.

**TABLE 3:** Multivariate analysis of clinicopathological characteristics affecting breast cancer-specific survival and disease-free survival in luminal A and triple-negative breast cancers.

Parameter	Breast cancer-specific survival		Disease-free survival	
	Hazard ratio (95% CI)	*p*-value	Hazard ratio (95% CI)	*p*-value
Luminal A subtype (*n* = 303)				
SASP, present *vs* absent	2.884 (1.204–6.909)	0.017	3.119 (1.731–5.618)	<0.001
LVI, present *vs* absent	-	-		-
Lymph node metastasis, present *vs* absent	-	-	1.982 (1.072–5.618)	0.029
Histologic grade, 3 *vs* 1 and 2			-	-
Tumor size, >2 cm *vs* ≤ 2 cm	11.031 (3.262–37.311)	<0.001	-	-
Triple-negative subtype (*n* = 247)				
SASP, present *vs* absent	0.355 (0.167–0.757)	0.007	0.437 (0.215–0.889)	0.022
LVI, present *vs* absent	-	-	4.66 (2.164–10.035)	<0.001
Lymph node metastasis, present *vs* absent	4.203 (1.891–9.342)	<0.001	-	-
iCD103 + lymphocyte**,** high (≥ 38/ mm^2^) *vs* low (< 38/ mm^2^)	0.412 (0.178–0.954)	0.038		
Stromal TILs,> 5% *vs* ≤ 5%	-	-	0.444 (0.222–0.888)	0.022

CI, confidence interval; iCD103 + lymphocyte, intraepithelial CD103-positive lymphocyte; LVI, Lymphovascular invasion; SASP, senescence-associated secretory phenotype; TILs, tumor-infiltrating lymphocytes.

## Discussion

In this study, SASP was found to be significantly associated with surrogate molecular subtype in IBC. SASP positivity was lowest in the luminal A subtype and highest in TNBC. This association concurs with a previous study, which reported that inhibition of ER in ER-expressing cells induced a senescence-like phenotype by increasing SA-β-Gal activity and decreasing retinoblastoma (RB) protein phosphorylation [39].

Regarding previous studies that investigated the expressions of senescence-associated markers in tumor tissues, Brezniceanu et al. [26] reported that HMGB1 is involved in the regulation of apoptosis and that its overexpression inhibits apoptosis and promotes tumor growth. These researchers also reported that apoptosis was induced by transfection of BAK into the RKO colon cancer cell line and the human and mouse kidney cell lines (203T and NRK1), but that cotransfection of BAK with HMGB1 significantly inhibited apoptosis. In addition, they investigated HMGB1 expression in normal and cancer tissues of the breast by Western blot and IHC and observed that HMGB1 expression was significantly higher in cancer tissues than in normal tissues. In a study on cotransfections of BAK and HMGB1 genes into NRK1 cells, Völp et al. [22] revealed HMGB1 expression inhibited BAK–induced apoptosis by blocking the activities of caspase-9 and–3 and found that this anti-apoptotic effect was due to inhibitors of apoptosis proteins (IAPs) that bind to and inhibit caspases. HMGB1 overexpression in the RKO cells increased nuclear factor kappa B (NFκB) activity and resulted in the overexpression of c-IAP2 (a target gene product of NFκB). In order to confirm these relationships in tissues, Volp et al. [22] examined the expressions of c-IAP2 and HMGB1 by IHC staining on TMAs of colon cancer and normal colon tissues from 29 patients. They found the expressions of c-IAP2 and HMGB1 were upregulated in 72.4% of colon cancer tissues, and that the expression of HMGB1 in tumor cell cytoplasm and nuclei was pronounced at tumor borders. Kim et al. [40] evaluated the immunohistochemical expressions of p16 and cyclin D1 in 224 cases and observed that p16/cyclin D1 indexes were significantly higher in HER2-positive and TNBC groups than in a luminal group and that this index was associated with poor prognosis. Pare et al. [28] investigated DCR2 expression in 1,032 tissues by IHC and reported DCR2 overexpression was associated with poor prognostic factors such as high histologic grade, ER negativity, PR negativity, and HER2 positivity. In addition, they found DCR2 overexpression was not related to age, tumor size, stage, or LVI, and reported that the DCR2 positive group tended to have poorer prognoses than the DCR2 negative group.

In the present study, SASP positivity was associated with poorer prognosis in the luminal A group and multivariate analysis showed SASP positivity independently predicted BCSS and DFS, and these results were broadly in-line with several previous studies. Kim et al. [41] investigated the role of cellular senescence in papillary thyroid carcinoma (PTC) and found that senescent tumor cells were present at the invasive borders of PTC and exhibited a higher invasive ability than non-senescent tumor cells by upregulated MMPs. They also demonstrated that senescent cancer cells lead the collective invasion through CXCL12/CXCR4 signaling and enhance collective LVI in thyroid cancer. Similar result was obtained in our study. LVI was more frequently observed in SASP-positive group than in SASP-negative group in luminal A .

Inflammatory cytokines secreted by senescent tumor cells can modulate tumor microenvironment. IL-6 was known to activate STAT3 and consequently increase MMP-2 expression and the metastatic ability of cancer cells in malignant melanoma [42]. Waugh and Wilson [43] reported IL-8 secreted by tumor cells plays an important role in the tumor microenvironment and observed that the expressions of the IL-8 receptors, CXCR1 and CXCR2, were elevated in cancer cells, endothelial cells, and tumor-infiltrating neutrophils and macrophages. The authors concluded IL-8 signaling increases cancer cell proliferation and survival, promotes angiogenesis by stimulating endothelial cells, and promotes the migration of neutrophils into tumor tissues and their activations. Vascular endothelial growth factor (VEGF, a SASP factor) is secreted by senescent cells and promotes cancer cell growth by promoting angiogenesis and facilitating access to growth factors [44]. Although we did not investigate the associations between SASP and tumor tissue concentrations of MMP, IL-6, or IL-8, we believe that the significantly higher frequency of LVI observed in SASP-positive tumors reflected increased tumor cell invasiveness induced by factors secreted by senescent tumor cells.

In tumor microenvironments, IL-6 signaling not only promotes proliferation and survival but also has anti-tumor effects due to inhibition of tumor growth through T-cell immune response. IL-6 promotes T cell migration to lymph nodes and tumor areas and can exhibit cytotoxic effects on tumor cells by activating T cells [45]. In the present study, SASP was significantly associated with high sTIL density and high iCD103 + lymphocyte levels. Although we did not measure IL-6 secreted by SASP-positive cells, we think that high sTIL density and high iCD103 + lymphocyte levels in SASP-positive tumors would be, in part, T cell migration to tumor site by IL-6 signaling by senescent cells. In contrast to that observed in the luminal A subtype, SASP positivity was associated with good prognosis in TNBC. In our previous studies, high sTIL density and high iCD103 + lymphocyte counts were good prognostic factors in a subset of s (e.g., TNBC) and iCD103 + lymphocyte infiltration was more closely related to prognosis than sTIL density [30]. In the present study, SASP was not related to sTIL density and iCD103 + lymphocyte count was significantly higher in SASP-positive cases in TNBC. These relationships may, in part, explain the better prognosis of SASP-positive tumors in TNBC. However, we suggest further study be conducted on relationships between biomarker expressions (ER, PR, and HER2) and biologic effects of SASP.

This study has several limitations. It is a single-center and retrospective study and immunohistochemical expression of SASP was observed in TMA tissues rather than in whole sections. Furthermore, we did not investigate SASP factors by placing focus on their functions. However, we conducted this study on a large cohort of primary IBCs with follow-up data.

Summarizing, immunohistochemically detected SASP in tissues was significantly associated with tumor aggressiveness (LVI, high histologic grade, and triple-negativity) and with immune microenvironments exhibiting high sTIL density and iCD103 + lymphocyte infiltration. In addition, the prognostic significance of SASP was found to be dependent on the surrogate molecular subtypes of breast cancer.

## Data Availability

The data that support the findings of this study are available from the corresponding author upon reasonable request. Requests to access the datasets should be directed to Young Kyung Bae, ykbae@ynu.ac.kr.
